# Tuberculin skin test and interferon‐γ release assays: Can they agree?

**DOI:** 10.1111/crj.13569

**Published:** 2022-12-16

**Authors:** João Almeida Santos, Raquel Duarte, Carla Nunes

**Affiliations:** ^1^ NOVA National School of Public Health, Public Health Research Centre Universidade NOVA de Lisboa Lisbon Portugal; ^2^ National Health Institute Dr. Ricardo Jorge Lisbon Portugal; ^3^ Centro Hospitalar de Vila Nova de Gaia Vila Nova de Gaia Portugal; ^4^ Faculdade de Medicina da Universidade do Porto Porto Portugal; ^5^ Comprehensive Health Research Center (CHRC) Universidade NOVA de Lisboa Lisbon Portugal

**Keywords:** interferon‐gamma release tests, latent tuberculosis infection, public health, tuberculin skin test

## Abstract

**Introduction:**

The diagnosis of latent tuberculosis infection (LTBI) relies largely on the tuberculin skin test (TST) or, more recently, on interferon‐gamma release assays (IGRA). Knowledge regarding these tests is essential to improve their usefulness in combating the tuberculosis epidemic.

**Objectives:**

To characterize the agreement between the IGRA and TST tests by determining the kappa coefficient (K) and agreement rate between these two tests in patients with active tuberculosis (TB).

**Methods:**

Retrospective cohort study conducted with data from active TB patients notified in the Portuguese Tuberculosis Surveillance System (SVIG‐TB), from 2008 to 2015. TST results were interpreted using a 5 mm (TST‐5 mm) and 10 mm (TST‐10 mm) cutoff. Kappa coefficient and agreement rate were calculated in order to evaluate the agreement between IGRA and TST (both cutoffs) test results.

**Results:**

A total of 727 patients with results for both tests were included in the study, of which 3.4% (*n* = 25) had HIV infection, 5.6% (*n* = 41) diabetes, 5.0% (*n* = 36) oncological diseases and 4.4% (*n* = 32) inflammatory diseases.

Of the 727 patients, 16.5% (*n* = 120) presented different outcomes between IGRA and TST‐5 mm, and 20.5% (*n* = 149) presented different outcomes between IGRA and TST‐10 mm. Kappa coefficient between IGRA and TST‐5 mm was 0.402 (*p* < 0.001) with an agreement rate of 83.5%. Between IGRA and TST‐10 mm, the kappa coefficient was 0.351 (*p* < 0.001), with an agreement rate of 79.5%. Patients with HIV infection, diabetes, oncologic diseases and inflammatory diseases presented a substantial agreement between IGRA and TST‐5 mm, while inflammatory diseases was the only variable that presented a substantial agreement between IGRA and TST‐10 mm.

**Conclusion:**

As both tests can present false‐negative results, the low level of agreement between the tests can potentially help identify more cases of LTBI if the two tests are used in parallel, with infections not detected by IGRA possibly being detected by the TST and vice versa.

## Graphical abstract

Tuberculin skin test (TST) and interferon‐gamma release assay (IGRA) are two assays based on cell‐mediated immunity (memory T cell response) available for screening potential cases of latent tuberculosis infections. 16.5% (IGRA/TST‐5 mm) and 20.5% (IGRA/TST‐10 mm) of the patients presented different outcomes in the tests. Combined use can help promote the identification of more cases of infection, with infections not detected by the IGRA potentially being detected by the TST and vice versa.

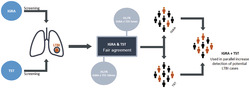

## 1 INTRODUCTION

About a quarter of the world's population is estimated to be infected with *Mycobacterium tuberculosis*.^1^ People with latent tuberculosis infection (LTBI) do not present clinical evidence of disease and are not infectious, but they are at risk of progressing to active tuberculosis (TB) at any time and, consequently, become a focus of transmission.^2^

For this reason, one of the components of the World Health Organization (WHO) End TB strategy is the screening of contacts and selected high‐risk groups in order to identify those who are at increased risk for developing TB disease and would benefit from prophylactic therapy.^3^

However, there is no diagnostic test that can unequivocally diagnose an *M. tuberculosis* infection. Instead, LTBI is diagnosed by indirect approaches that provide immunological evidence of host sensitization to *M. tuberculosis* antigens.^4^ Currently, there are two assays based on cell‐mediated immunity (memory T cell response) available for identification of LTBI cases: the tuberculin skin test (TST) and the gamma interferon release assays (IGRA).^5^ Neither diagnostic test can accurately differentiate active disease from latent infection nor reflect the risk of progression from latent to active TB, but they can facilitate diagnostic decisions when used in conjunction with other clinical and epidemiological information.^4^, ^6^ Consequently, knowledge about these tests is essential to improve their usefulness in combating TB epidemics. Whether it is in defining which test to use in risk groups, such as people living with HIV, or realizing whether the combined use of the tests could mean added value information. Therefore, the aim of the study was to ascertain the agreement between the IGRA and TST tests by determining the kappa coefficient (K) and agreement rate between these two tests in patients with active TB.

## 2 MATERIAL AND METHODS

### 2.1 Study design and data source

Retrospective cohort study regarding data from pulmonary and extrapulmonary TB cases notified in the Portuguese Tuberculosis Surveillance System (SVIG‐TB), from 2008 to 2015. Patients notified in the database were diagnosed based on laboratory confirmed TB and/or based on clinical and radiological findings and a favourable TB treatment response.

### 2.2 Study data

All TB cases notified in the SVIG‐TB database with an IGRA and TST test were included in the study. The IGRA test used in the patients enrolled in the study was the QuantiFERON‐TB Gold In‐Tube (Qiagen). Several clinical and sociodemographic variables were included in the analysis (Table 1).

### 2.3 Statistical analysis

TST induration measurements were interpreted as positive using two cutoff points: ≥5 mm (TST‐5 mm) and ≥10 mm (TST‐10 mm).

Descriptive statistics was used to characterize the cases included in the study. Kappa coefficient (k) was used to evaluate the agreement between IGRA and TST test results, with K values classified as slight (k < 0.20), fair (0.21 < k < 0.40), moderate (0.41 < k < 0.60), substantial (0.61 < k < 0.80) and almost perfect (0.81 < k < 1.0).^7^ The agreement rate between the two tests was also calculated (sum of cases in agreement/total number of cases multiplied by 100). Statistical analyses were performed using SPSS®, ver. 23.

## 3 RESULTS

Of the 727 patients included in the study, half were female (*n* = 365, 50.2%), with a mean (± standard deviation) age of 46.7 (±20.3) years (Table 1). Portugal was the main country of origin (*n* = 642; 88.3%), followed by Portuguese speaking countries such as Angola (*n* = 21, 2.9%) and Cabo Verde (*n* = 13, 1.8%).

**TABLE 1:** Sociodemographic and clinical characteristics of the patients (*n* = 727) included in the analysis and the IGRA and TST results stratified according to these characteristics

Variables		Patients	IGRA results		TST‐5 mm results		TST‐10 mm results	
		n (%)	Positive	Negative	Positive	Negative	Positive	Negative
Sex	Male	362 (49.8)	304	58	304	58	278	84
	Female	365 (50.2)	295	70	311	54	292	73
Age	≤15	48 (6.6)	37	11	35	13	31	17
	16–64	523 (71.9)	431	92	458	65	423	100
	≥65	156 (21.5)	131	25	122	34	116	40
Comorbidities	HIV infection	25 (3.4)	18	7	16	9	15	10
	Diabetes	41 (5.6)	32	9	31	10	27	14
	Oncologic diseases	36 (5.0)	23	13	23	13	20	16
	Inflammatory diseases	32 (4.4)	20	12	24	8	21	11
Substance abuse	Alcohol abuse	31 (4.3)	28	3	27	4	25	6
	Drug abuse	32 (4.4)	27	5	31	1	31	1
Site of infection	Pulmonary	343 (47.2)	275	68	292	51	270	73
	Extrapulmonary	384 (52.8)	324	60	323	61	300	84

Abbreviations: IGRA, interferon‐gamma release assays; TST, tuberculin skin test.

### 3.1 IGRA and TST agreement

**FIGURE 1:**
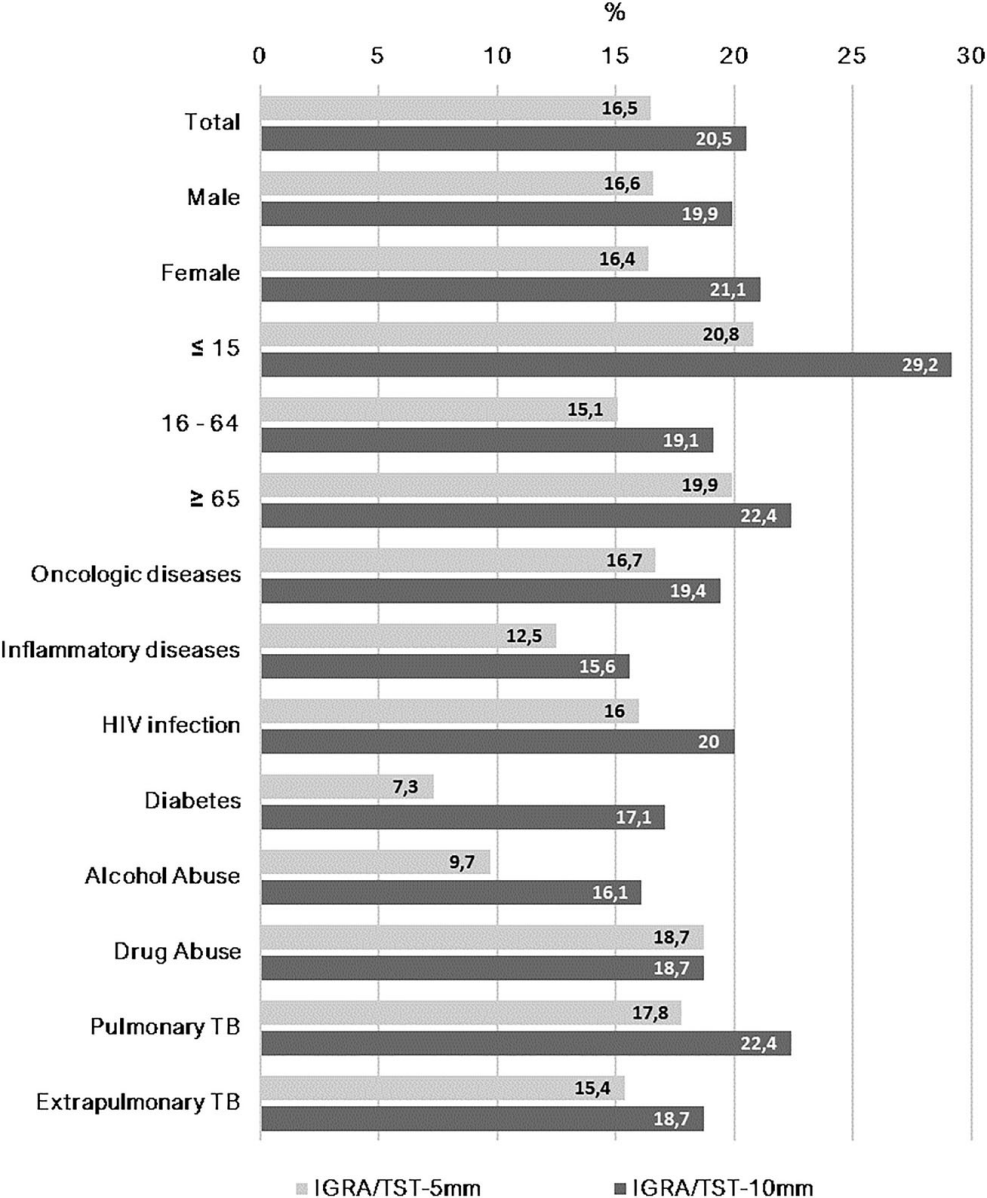
Proportion of patients with different outcomes between IGRA and TST‐5 mm and between IGRA and TST‐10 mm

The level of agreement between IGRA and TST‐5 mm, determined through the kappa coefficient, was 0.402 (*p* < 0.001) with an agreement rate of 83.5%, indicating a fair agreement between the tests. 16.5% (*n* = 120) of the patients showed different outcomes between the two tests (Figure 1). 8.3% (*n* = 60) and 75.2% (*n* = 547) of the patients presented a negative and positive result in both tests, respectively.

With the TST‐10 mm, the agreement between the tests was 0.351 (*p* < 0.001), with an agreement rate of 79.5%, indicating a fair agreement between the tests. 20.5% (*n* = 149) of the patients showed different outcomes between the IGRA and TST‐10 mm tests (Figure 1).

9.4% (*n* = 68) and 70.2% (*n* = 510) of the patients presented a negative and positive result in both tests, respectively.

IGRA and TST‐5 mm presented a substantial agreement in patients with HIV infection (k = 0.635), diabetes (k = 0.795), oncologic diseases (k = 0.639) and inflammatory diseases (k = 0.714) but showing slight to moderate agreement in the remaining variables (Table 2).

**TABLE 2:** Agreement between IGRA and TST (TST‐5 mm and TST‐10 mm) in TB cases

Variables		IGRA and TST‐5 mm				IGRA and TST‐10 mm			
		k	*p*‐value	Interpretation	Agreement Rate	k	*p*‐value	Interpretation	Agreement rate
Sex	Male	0.384	<0.001	Fair	83.4	0.374	<0.001	Fair	80.1
	Female	0.419	<0.001	Moderate	83.6	0.33	<0.001	Fair	78.9
Age	≤15	0.446	0.002	Moderate	79.2	0.307	0.026	Fair	70.8
	16–64	0.411	<0.001	Moderate	84.9	0.362	<0.001	Fair	80.9
	≥65	0.356	<0.001	Fair	80.1	0.329	<0.001	Fair	77.6
Comorbidities	HIV infection	0.635	0.001	Substantial	84	0.561	0.004	Moderate	80
	Diabetes	0.795	<0.001	Substantial	92.7	0.585	<0.001	Moderate	82.9
	Oncologic diseases	0.639	<0.001	Substantial	83.3	0.599	<0.001	Moderate	80.6
	Inflammatory diseases	0.714	<0.001	Substantial	87.5	0.661	<0.001	Substantial	84.4
Substance abuse	Alcohol abuse	0.518	0.003	Moderate	90.3	0.362	0.029	Fair	83.9
	Drug abuse	−0.055	0.662	Slight	81.3	−0.055	0.662	Slight	81.3
Site of infection	Pulmonary	0.382	<0.001	Fair	82.2	0.313	<0.001	Fair	77.6
	Extrapulmonary	0.421	<0.001	Moderate	84.6	0.389	<0.001	Fair	81.3

Abbreviations: IGRA, interferon‐gamma release assays; k, kappa coefficient; TST, tuberculin skin test.

Inflammatory diseases (k = 0.661; *p* < 0.001) was the only variable that presented a substantial agreement between IGRA and TST‐10 mm results, with the rest of the variables associated with slight to moderate agreement (Table 2).

## 4 DISCUSSION

IGRA and TST presented only a slight level of agreement, with 16.5% of the patients showing different outcomes between IGRA and TST‐5 mm and 20.5% between IGRA and TST‐10 mm. The level of agreement was lower with TST‐10 mm than TST‐5 mm, globally and in the majority of the clinical and sociodemographic variables studied (only in patients with a drug problem was the level of agreement identical).

This level of agreement is in line with the results obtained in a meta‐analysis^8^ that compared TST and IGRA results of 24 studies and obtained an overall kappa value of 0.28 (95%CI: 0.22–0.35), which reflected the fact that almost a third of the results were discordant. The level of agreement between the tests appears to be dependent on different factors such as BCG vaccination, incidence of infection or age,^8^ which leads to some studies showing a moderate^9^ to good^10^ agreement between the two tests. However, the meta‐analysis performed by Lamberti et al.^8^ only included studies carried out in healthcare workers, whereas in our study, we analysed the performance of the tests in patients with a diagnosis of TB and, furthermore, with different risk factors that could influence negatively the IGRA and TST results (e.g., HIV infection). Thus, adding knowledge about the performance of these tests in population groups where they will be most needed.

In our study, IGRA and TST‐5 mm presented a good agreement in patients with comorbidities: HIV infection, diabetes, oncologic and inflammatory diseases. These comorbidities can cause a reduction in the sensitivity of at least one of the tests,^4^, ^11^, ^12^, ^13^ as shown by the lower proportion of positive results obtained in a previous study that included these patients.^14^ This reduction in sensitivity apparently caused the outcomes obtained by the two tests to have a greater degree of agreement (more IGRA negative/TST negative results).

These results seem to suggest that the combined use of the two tests could help to promote the identification of more cases of infection than if used separately. This could be especially useful when detecting LTBI among high‐risk groups,^15^ such as people living with HIV.

## CONFLICT OF INTEREST

Fundação para a Ciência e Tecnologia provided financial support for this research [Grant: PTDC/SAU‐PUB/31346/2017] but had no involvement in the research itself.

The authors report no conflicts of interest.

## ETHICS STATEMENT

This study was a secondary data analysis of the fully anonymized SVIG‐TB dataset; therefore, ethics committee approval and informed consent were not required. The study received approval from the research ethics committee of the Administração Regional de Lisboa e Vale do Tejo (Reference 3514/CES/2019).

## AUTHOR CONTRIBUTIONS

All authors made substantial contributions to the conception and design of the study and all gave the final approval of the version to be submitted. João Almeida Santos and Carla Nunes developed the design of the study. João Almeida Santos implemented the study, carried out the analysis and interpretation of the data and drafted the original manuscript. Carla Nunes and Raquel Duarte collaborated in the analysis and critical revision of the manuscript.

## Data Availability

The dataset supporting the conclusions of this article was obtained through the National Tuberculosis Surveillance System (SVIG‐TB) and is not publicly available. The SVIG‐TB is overseen by the Directorate General for Health (DGS) which grants access to the fully anonymized dataset for epidemiological studies, and it is possible to request the data used in this specific study upon reasonable request.

## References

[1] Houben R , Dodd P . The global burden of latent tuberculosis infection: a re‐estimation using mathematical modelling. PLoS Med. 2016;13(10):1‐13. doi:10.1371/journal.pmed.1002152 PMC5079585 27780211

[2] Dutta NK , Karakousis PC . Latent tuberculosis infection: myths, models, and molecular mechanisms. Microbiol Mol Biol Rev. 2014;78(3):343‐371. doi:10.1128/mmbr.00010-14 25184558 PMC4187682

[3] WHO . Implementing the end TB strategy: The essentials. World Health Organization; 2015.

[4] Pai M , Denkinger C , Kik SV , et al. Gamma interferon release assays for detection of mycobacterium tuberculosis infection. Clin Microbiol Rev. 2014;27(1):3‐20. doi:10.1128/CMR.00034-13 24396134 PMC3910908

[5] Getahun H , Matteelli A , Chaisson RE , Raviglione M . Latent mycobacterium tuberculosis infection. N Engl J Med. 2015;372(22):2127‐2135. doi:10.1056/NEJMra1405427 26017823

[6] Salgame P , Geadas C , Collins L , Jones‐López E , Ellner JJ . Latent tuberculosis infection—revisiting and revising concepts. Tuberculosis. 2015;95(4):373‐384. doi:10.1016/j.tube.2015.04.003 26038289

[7] Landis JR , Koch GG . The measurement of observer agreement for categorical data. Biometrics. 1977;33(1):159. doi:10.2307/2529310 843571

[8] Lamberti M , Uccello R , Monaco MGL , et al. Tuberculin skin test and Quantiferon test agreement and influencing factors in tuberculosis screening of healthcare workers: a systematic review and meta‐analysis. J Occup Med Toxicol. 2015;10(1):2. doi:10.1186/s12995-015-0044-y 25670962 PMC4323208

[9] Wlodarczyk M , Rudnicka W , Janiszewska‐Drobinska B , et al. Interferon‐gamma assay in combination with tuberculin skin test are insufficient for the diagnosis of culture‐negative pulmonary tuberculosis. PLoS ONE. 2014;9(9):e107208. doi:10.1371/journal.pone.0107208 25221998 PMC4164613

[10] Machado A , Emodi K , Takenami I , et al. Analysis of discordance between the tuberculin skin test and the interferon‐gamma release assay. Int J Tuberc Lung Dis. 2009;13(4):446‐453. 19335949

[11] Choi JC , Jarlsberg LG , Grinsdale JA , et al. Reduced sensitivity of the QuantiFERON® test in diabetic patients with smear‐negative tuberculosis. Int J Tuberc Lung Dis. 2015;19(5):582‐588. doi:10.5588/ijtld.14.0553 25868028

[12] Lange B , Vavra M , Kern WV , Wagner D . Indeterminate results of a tuberculosis‐specific interferon‐? Release assay in immunocompromised patients. Eur Respir J. 2010;35(5):1179‐1182. doi:10.1183/09031936.00122109 20436175

[13] Lewinsohn DM , Leonard MK , Lobue PA , et al. Official American Thoracic Society/Infectious Diseases Society of America/Centers for Disease Control and Prevention clinical practice guidelines: diagnosis of tuberculosis in adults and children. Clin Infect Dis. 2017;64(2):e1‐e33. doi:10.1093/cid/ciw694 27932390

[14] Santos JA , Duarte R , Nunes C . Host factors associated to false negative and indeterminate results in an interferon‐γ release assay in patients with active tuberculosis. Pulmonology. 2020;26(6):353‐362. doi:10.1016/j.pulmoe.2019.11.001 31843341

[15] Heuvelings CC , Vries SG , Grobusch MP . Tackling TB in low‐incidence countries: improving diagnosis and management in vulnerable populations. Int J Infect Dis. 2017;56:77‐80. doi:10.1016/j.ijid.2016.12.025 28062228

